# Eye Gaze Patterns of Decision Process in Prosocial Behavior

**DOI:** 10.3389/fnbeh.2020.525087

**Published:** 2020-10-22

**Authors:** Anastasia Peshkovskaya, Mikhail Myagkov

**Affiliations:** ^1^Laboratory of Experimental Methods in Cognitive and Social Sciences, Tomsk State University, Tomsk, Russia; ^2^Mental Health Research Institute, Tomsk National Research Medical Center, Russian Academy of Sciences, Tomsk, Russia; ^3^Institute of Education, National Research University Higher School of Economics, Moscow, Russia; ^4^University of Oregon, Eugene, OR, United States

**Keywords:** visual perception, eye tracking, decision making, prosocial behavior, gaze, eye movements, cooperation, decision strategies

## Abstract

Understanding human behavior remains a grand challenge across disciplines. We used eye tracking to investigate how visual perception is associated with a strategic behavior in the decision process. Gaze activity and eye movement patterns were measured in 14 human participants with different decision strategies. We also employed a social domain to force strategic behavior. We find that social interaction significantly improves the level of cooperation, prosocial decisions, and overall cooperative strategy in experiment participants. Gaze behavior in individuals with a cooperative strategy is characterized by a greater number of fixations and frequent gaze returns to the scanned areas. On the contrary, individuals with a non-cooperative strategy approach decision-making task stimuli in a distinct way with long-duration fixations and a low number of gaze returns to the areas already scanned. Social domain, which enhances cooperation and prosocial behavior, makes participants more attentive to the task stimuli in our experiments. Moreover, prolonged gaze at the area of cooperative choice testifies in favor of the cooperative decision.

## Introduction

Eye tracking is widely used to study cognition based on visual perception (Kahneman and Beatty, 1966; Schwedes and Wentura, 2012; Dalmaso et al., 2017; Eckstein et al., 2017). Decision making has been a subject of research interest since at least the 1970s (Payne et al., 1978, 1993; Ford et al., 1989), but eye tracking technology has been used only recently (Glaholt et al., 2010; Glaholt and Reingold, 2011; Krajbich and Rangel, 2011; Gidlöf et al., 2013; Peshkovskaya et al., 2017). As the theoretical framework for understanding human decision making, a social dilemma game is employed frequently as it represents human interactions in a variety of settings. In particular, the prisoner’s dilemma is the most commonly employed game in behavioral and psychological studies (Kieslich and Hilbig, 2014; Peshkovskaya et al., 2018; Haesevoets et al., 2020) due to its potential to interpret the emergence and survival of cooperative behavior (Doebeli and Hauert, 2005; Perc and Wang, 2010; Wang et al., 2012). It is known that the prisoner’s dilemma game player has two strategies: cooperation and non-cooperation, the latter commonly named defection. In this research, we used eye tracking technology and the prisoner’s dilemma together to investigate decision making and features of visual perception associated with strategic experience, particularly with prosocial, cooperative strategy. We also assume that social domain could contribute to cooperative strategy and shape prosocial behavior (Shigaki et al., 2013; Peshkovskaya et al., 2019a). Thereby, we focused our study to identify specific features of eye movements for cooperative and non-cooperative strategies.

A large number of studies suggest a tight coupling between eye movements, cognition, and behavioral peculiarities (Sharot et al., 2008; Isham and Geng, 2013; Foulsham and Lock, 2015; Gillath et al., 2017). Works on one-shot games reveal that players approach the visual stimuli selectively and focus their attention on certain areas only. For example, they have more gaze fixations on the maximum and minimum payoffs (Hristova and Grinberg, 2005; Devetag et al., 2016). Furthermore, certain perception patterns could be based on strategic experience. The research by Polonio et al. (2015) shows that equilibrium strategy is accompanied by a certain eye movement pattern: an individual looks sequentially at his or her payoffs first, then at the game partner’s payoffs, integrates them, and finally looks at his or her own expected payoffs if the choice is equal. Individuals who avoid equilibrium strategy mainly pay attention only to their payoffs (Polonio et al., 2015).

It is also shown that actions committed under the influence of the social environment occur faster (Nishi et al., 2016). Cooperative decisions are made faster in a cooperative environment. Decisions not to cooperate are made more quickly in a non-cooperative environment. In other words, the environment influences behavior and contributes to the maintenance of a certain strategy.

Interestingly, gaze fixations and gaze time as well as decision time can potentially forecast the behavior of people with a different type of social value orientation. Altruists look at the opponent’s payoffs. Individualists look at their own (Fiedler et al., 2013). It also takes a long time for individuals with cooperative and competitive social value orientation to make decisions: their number of fixations is higher, and attention is paid to both their payoffs and those of the opponent. Therefore, it is assumed that there is a link between parameters of eye movements, decision time, and social preferences according to which people are more or less inclined to cooperate. However, the efficiency of predicting how type of social value orientation impacts cooperation level is limited.

In addition, there is a large amount of evidence that social factors have an impact on decision making (Jiang et al., 2016; Giacomantonio et al., 2018). Cooperative, prosocial behavior can be generated through social interaction between members of the group, accompanied by identification with the group, which shapes an added value of collective interaction (Tajfel et al., 1971; Dasgupta, 2004; Kozitsin et al., 2019, 2020; Myagkov et al., 2019; Peshkovskaya et al., 2019b). Generally, prosociality consists of a broad constellation of attitudes, values, and behaviors that involve cooperating with others (Wilson, 2007). The development of prosocial behavior is foundational for the ongoing existence of any community of people (Baumsteiger, 2019; Thielmann et al., 2020). We use this theoretical and methodological approach to generate cooperative behavior in our experiments.

In this article, we present results of 14 experiments conducted at Tomsk State University (Russia). One participant in each experiment was equipped with eye-tracking glasses (ETG). The experiment was conducted in three stages: an Anonymous stage against randomly chosen partners; a Social Interaction stage, consisting of communication between participants and further group formation; and a Group stage, in which participants played with partners within the newly formed groups. This laboratory model combines the classic social psychology minimal group paradigm with group manipulations that cause a sense of social attachment (Dasgupta, 2004). The use of this model in the experiments reveals that social interaction leads to higher levels of cooperation and its persistence in participants within social group (Peshkovskaya et al., 2018, 2019a).

The study was aimed to answer the following questions:

(1) Are there any differences in gaze behavior in individuals with different decision strategies and level of cooperation?
(2) How do features of gaze behavior associate with decision strategy?
(3) Do the features of strategic-based gaze behavior vary under the influence of social interaction?

## Materials and Methods

### Participants

Fourteen experiments were conducted with the mobile eye-tracking system. Participants (*N* = 168) were recruited as volunteers through the social network VK (vk.com). All the experiments were conducted in groups of 12 participants. Only one participant in every experiment was equipped with the ETG. Therefore, the sample of the current study includes data from 14 individuals: 7 women and 7 men between the ages of 20 and 40 years (*M* = 23.7, SD = 6.2) living in Tomsk, Russian Federation.

The study procedures involving human participants were approved by Tomsk State University Human Subjects Committee and adhered to the tenets of the Declaration of Helsinki. The methods in the study are in accordance with relevant guidelines, and a written informed consent was obtained from all participants. Neither of the experiments reported in this article was formally preregistered. Experimental data are readily available on Harvard Dataverse (Peshkovskaya, 2020).

### Prisoner’s Dilemma

We use the prisoner’s dilemma game (hereinafter PD) to study features of the decision-making process. PD is a game for two players. Each of two players in the PD game has two strategies: Cooperation (Up or Left) or Defection (Down or Right). Two players in the standard PD are offered the same points, R for Cooperation and a smaller gain, P for Defection. If one of the players cooperates and another defects, the cooperator gains a smaller reward, T, but the defector takes a larger reward, S. Thus, there is a ratio between prizes T > R > P > S (Table 1). Defection is more profitable than Cooperation in any partner’s choice, but mutual Cooperation is more profitable for both than mutual Defection. The Nash equilibrium corresponds to mutual Defection (P, P), but the participants try to establish mutual Cooperation (R, R).

**TABLE 1:** Prisoner’s dilemma payoffs.

**Payoffs**	***Cooperation***	***Defection***
*Cooperation*	R, R	S, T
*Defection*	T, S	P, P

*For the experiments considered in this article, the parameters of the PD were set as *R* = 5, *P* = 1, *S* = 0, and *T* = 10 (10 > 5 > 1 > 0).*

### Research Design

#### Procedure

Twelve participants were invited to a computer classroom where they completed the participant consent form. One participant of the 12 was equipped with mobile ETG v.1.8 by SensoMotoric Instruments GmbH. The inclusion criterion for participants with ETG was normal uncorrected vision. The exclusion criteria were (1) a medical history of any vision impairment or (2) myopia. A participant was equipped with ETG from the beginning to the end of the experiment. All the participants received written and verbal instructions for the PD game. Experimenters announced that all points that the participants win in the PD game will be converted into real money.

#### Anonymous Stage

The participant with ETG was subjected to a 1-point calibration accuracy test. Then, all the experiment participants proceeded to the PD game (20 trials). A specialized tool to design and carry out group experiments, *z*-Tree, developed at the University of Zurich, was used (Fischbacher, 2007). The PD game interface design was identical for all experiment participants and was used in all experiments.

During the PD game, participants were able to move to the next trial only after all of them had made their choices. No one knew who their partner was. Moreover, pairs of participants changed randomly in each game trial. Trial results and the overall game results were displayed on a monitor after each trial. After the game was completed, calibration verification was conducted and the ETG participant removed the ETG.

#### Social Interaction Stage

Social factors have an impact on decision making (Peshkovskaya et al., 2019a; Haesevoets et al., 2020). Based on this, we used a social domain to force a subject’s strategic behavior in our experiments. Participants were involved in a social interaction through the communication and group formation. This laboratory model combines the classic social psychology minimal group paradigm with group manipulations that cause a sense of social attachment and shape prosocial behavior (Nishi et al., 2016; Peshkovskaya et al., 2018).

Communication between participants was shaped by an ice-breaking exercise (Peshkovskaya et al., 2017). Participants memorized each other’s names and gave a short self-report on their personal characteristics, hobbies, etc. Next, two captains were voluntarily selected. Then, participants were voluntary divided into two groups (six members each). After that, members of each newly formed group were asked to find 3 to 5 characteristics that were common to all group members and to choose a name for their group.

#### Group Stage

Participants took their seats at the computers. They were instructed that they would be asked to play the PD game again, however, this time their partner would be a random member from their newly formed group of six people. The participant who was equipped with the ETG at the Anonymous stage put on the ETG and completed the calibration accuracy test once again. Then all the participants proceeded to the PD game, which consisted of 22 trials. The result of each trial and the total personal game results were displayed on the participants’ monitors after each game trial.

Distribution of roles (selection of rows or columns) during the Anonymous and the Group stages occurred randomly.

**FIGURE 1:**
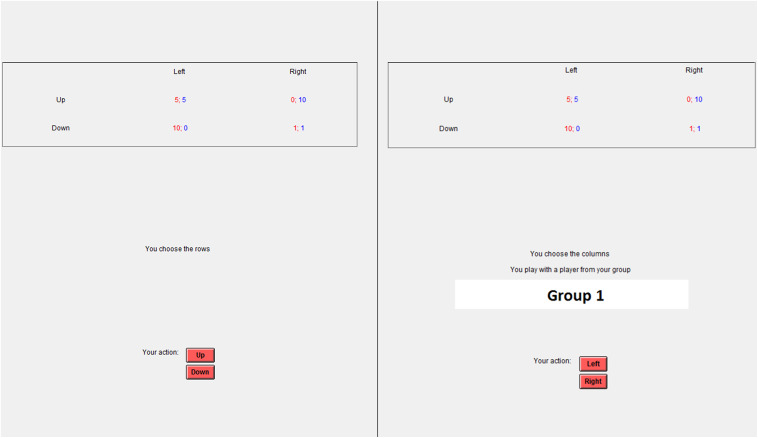
Example of stimuli. PD game interface at the Anonymous stage **(left)** and Group stage **(right)**.

Group names, which were created by participants during the Social Interaction Stage were displayed on monitors at the Group stage (Figure 1).

#### Eye Movement Parameters Processing and Extracting

BeGaze software was used to process, aggregate, and quantitatively analyze the eye-movement data. Semantic gaze mapping, which allowed us to create and modify reference views and mapping gaze data from scene videos to reference views, was used for processing and aggregating each ETG participant’s eye-movement data in each game trial.

**FIGURE 2:**
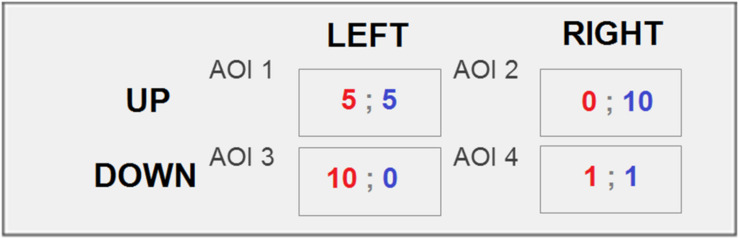
Marked AOI for the PD game matrix. Figure represents the payoff matrix of the PD. Every cell is an AOI.

The cells of the PD game payoff matrix were used in eye-movement data collection and analysis as the areas of interest (hereinafter AOI; Figure 2).

Eye movement parameters on each game trial were calculated as the key performance indicators (KPIs) with BeGaze software:

Revisits – amount of gaze returns to the already scanned area.
Fixation Count – number of gaze fixations on the certain area per second.
Dwell Time – total gaze time for the certain area (for example, the AOI area) as a percentage of the time the whole area was displayed.
Fixation Time – time of gaze fixation in the certain area as a percentage of the total gaze time.
Average Fixation Duration – the mean value of fixation time in the specified area.

Then, quantitative eye movement parameters based on the KPI of each ETG participant on each experimental stage and each game trial were extracted from BeGaze and statistically processed by StatSoft Statistica v. 10.

### Decision Making Strategies: Defectors and Cooperators

The data on cooperation varied significantly among the participants. We used cooperation data from the first experimental stage (Anonymous stage) as a baseline for cooperation level identification. We found that 7 out of 14 ETG participants showed a low cooperation level (11.6% cooperative decisions on average). They preferred to defect significantly more than to cooperate in game trials (*Z* = 2.485, *p* = 0.013, and Mann–Whitney *U*-test). The other 7 participants made 37.7% cooperative decisions on average. We defined their strategy as cooperative.

## Results

Analysis of variance was carried out to explore the association between experimental variables and gaze behavior. The effects of experimental stage (Anonymous versus Group stage), participant’s game role (choice between rows versus columns), decision type (cooperative versus non-cooperative) as well as combined effect of these variables were investigated (Table 2).

**TABLE 2:** Multivariate tests of significance.

	**Value**	***F***	**Effect – df**	**Error – df**	***p*-value**
Intercept	0.038	1663.74	6	390	0.0000001
Stage	0.909	6.55	6	390	0.000001
Role	0.986	0.95	6	390	0.457
Decision	0.951	3.33	6	390	0.003
Stage*Role	0.988	0.82	6	390	0.557
Stage*Decision	0.977	1.51	6	390	0.174
Role*Decision	0.991	0.62	6	390	0.713
Stage* < *cps*:*it* > *Role* < /*cps*:*it* > *Decision	0.987	0.85	6	390	0.534

*Sigma-restricted parameterization. We find that decision strategy as well as experimental stages are significant for gaze behavior variations.*

### Gaze Behavior and Decision Strategy

Participants using a defection strategy have a significantly smaller number of revisits to the already scanned payoff matrix areas, fewer fixations, and longer duration of fixation than Cooperators at the Anonymous (baseline) stage.

The number of gaze fixations for Defectors was lower than for Cooperators (*Z* = 3.534, *p* = 0.0004, and Mann–Whitney *U*-test); however, the average duration of fixation is higher in Defectors (*Z* = -3.054, *p* = 0.002, and Mann–Whitney *U*-test). In addition, Defectors showed a lower number of revisits to the areas in the payoff matrix scanned at least once at the Anonymous stage (*Z* = 3.402, *p* = 0.0007, and Mann–Whitney *U*-test; Table 3).

**TABLE 3:** Differences in Cooperators’ and Defectors’ gaze behavior.

	**Cooperators**		**Defectors**		***Z***	***p*-value**
	**Mean**	**Median**	**Mean**	**Median**		
Revisits	1.18	1.00	0.38	0.00	3.402	0.0007
Fixation count	3.24	2.00	1.85	1.00	3.534	0.0004
Average fixations duration, ms	161.73	155.60	241.28	194.05	–3.054	0.002

*The table illustrates the differences between Cooperators and Defectors in the parameters of eye movements when studying the payoff matrix in the Anonymous stage, Mann–Whitney *U*-test.*

In addition, we assumed that social factors have an impact on decision making. Therefore, we used a social domain to force strategic behavior in our experiments. We aimed to generate prosocial behavior through interaction between participants to explore how strategic and gaze behavior were changed under social influence.

**FIGURE 3:**
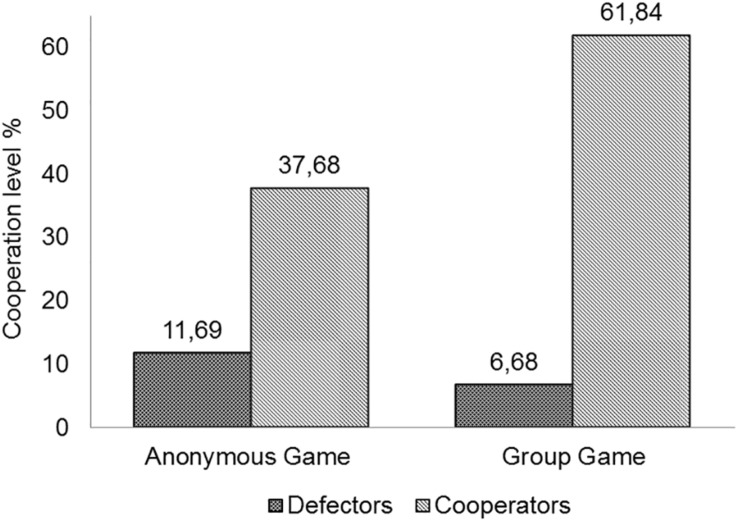
Mean cooperation level in the participants with different decision-making strategies.

First, we found that cooperation significantly increased in the Cooperators (from 37.68% to 61.84%, *p* = 0.0003, sign test) and diminished in Defectors (from 11.69% to 6.68% *p* = 0.006, sign test; Figure 3).

Second, eye-movement analysis indicated typical tendencies in gaze behavior of Cooperators and Defectors through the experimental stages. The eye-movement parameters of Cooperators at the Group stage were characterized by a greater number of fixations (*Z* = 2.483, *p* = 0.013, and Mann–Whitney *U*-test) and revisits to the areas already scanned (*Z* = 2.167, *p* = 0.030, and Mann–Whitney *U*-test) in comparison with Defectors. At the same time, Defectors had more prolonged average fixation duration than Cooperators (*Z* = −4.247, *p* = 0.00002, and Mann–Whitney *U-*test; see Supplementary Table 1).

Interestingly, this finding is consistent with Cooperators’ and Defectors’ gaze behavior at the Anonymous stage. Therefore, we suggest that Cooperators paid more attention to the stimuli and demonstrated a more “careful” approach in visual behavior before making their final decision, whereas Defectors consistently made longer gaze fixations.

### Eye Catchers for Individuals With Different Decision Strategies

Our next task was to investigate how much attention participants with different strategies paid to each element of the PD payoff matrix and which stimuli elements (AOI) were their eye catchers.

Importantly, we found no differences between total time spent by both Cooperators and Defectors in gazing in the payoff matrix at both experimental stages: 7.9 versus 7.8 for Dwell time and 6.65 versus 7.4 for Fixation Time at the Anonymous stage consequently; as well as 10.6 versus 11.4 for Dwell time and 10.45 versus 11.4 for Fixation Time at the Group stage consequently; (medians, Mann–Whitney *U*-test, all *P*s > 0.05; Table 4).

**TABLE 4:** Time parameters of eye movements in participants with a different decision strategy during experimental stages.

**Stage**	**Time parameters**	**Cooperators**		**Defectors**		***Z***	***p*-value**
		**Mean**	**Median**	**Mean**	**Median**		
Anonymous stage	Dwell time, %	9.18	7.9	12.82	7.80	–0.754	0.451
	Fixation time, %	8.03	6.65	11.95	7.40	–1.221	0.222
Group stage	Dwell time, %	13.20	10.60	11.85	11.40	–0.056	0.955
	Fixation time, %	11.88	10.45	11.39	11.40	–0.406	0.685

Clearly, Defectors and Cooperators watched the payoff matrix with equal timing, but as we previously show, in distinct ways. However, which cells of the payoff matrix attracted the largest share of their attention?

**FIGURE 4:**
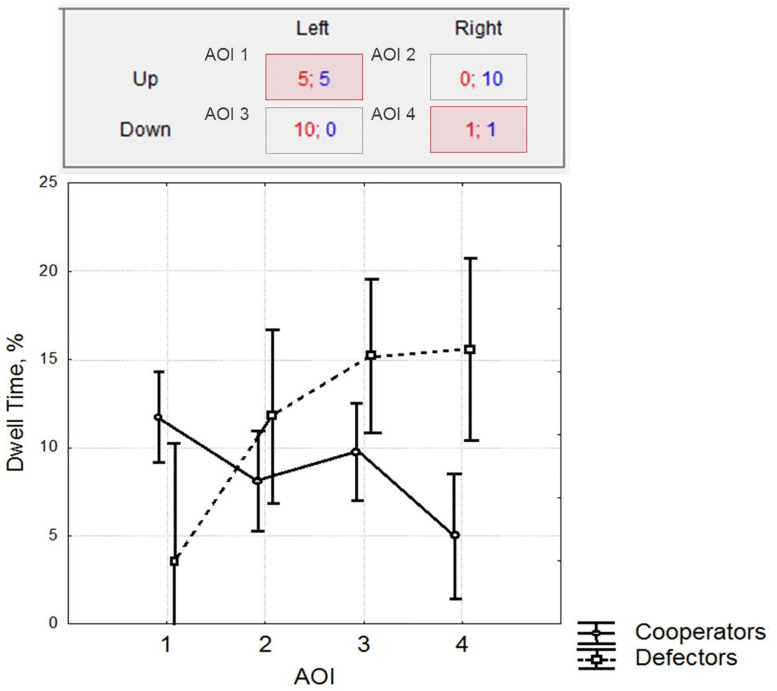
Statistically significant differences highlighted for area AOI 1 (cooperative decision) and AOI 4 (non-cooperative decision) in Cooperators and Defectors (Kruskall–Wallis test, all *P*s < 0.05). The figure represents the differences in Dwell time for AOI between Cooperators and Defectors.

We obtained significant differences in Defectors’ and Cooperators’ gaze time for areas AOI 1 (cell with payoffs 5 to 5, corresponding to cooperative decision) and AOI 4 (cell with payoffs 1 to 1, corresponding to non-cooperation; Figure 4).

Obviously, participants with a cooperative strategy paid more attention to the area of cooperative decision and spent less time looking at the non-cooperative matrix cell. This finding confirms the evidence of certain behavioral tendency in Cooperators to be less interested in non-cooperative decision outcomes than Defectors.

### Gaze Behavior Dynamics

To explore the changes in gaze behavior in two experimental stages, eye movement of Cooperators and Defectors were consistently compared. Changes in Cooperators’ Fixation Count, Dwell Time, and Fixation Time were recorded from the Anonymous Game stage to the Group Game stage (Tables 5, 6).

**TABLE 5:** Cooperators’ gaze behavior dynamics through the experimental stages.

	**Anonymous stage**		**Group stage**		***Z***	***p*-value**
	**Mean**	**Median**	**Mean**	**Median**		
Revisits	1.18	1.00	0.700	0.00	1.949	0.051
Fixation count	3.24	2.00	2.350	2.00	2.242	0.025
Average fixation duration, ms	161.73	155.60	168.72	161.25	–0.374	0.708
Dwell time, %	9.18	7.90	13.203	10.60	–3.088	0.002
Fixation time, %	8.03	6.65	11.884	10.45	–3.545	0.0004

*The table illustrates the changes in parameters of eye movements in Cooperators during the experimental stages: Anonymous and Group stage, Wilcoxon matched pairs test.*

**TABLE 6:** Defectors’ gaze behavior dynamics through the experimental stages.

	**Anonymous stage**		**Group stage**		***Z***	***p*-value**
	**Mean**	**Median**	**Mean**	**Median**		
Revisits	0.38	0.00	0.24	0.00	0.425	0.671
Fixation count	1.85	1.00	1.56	1.00	0.560	0.576
Average fixation duration, ms	241.28	194.05	242.95	256.00	–2.023	0.043
Dwell time, %	12.82	7.80	11.85	11.40	–1.126	0.260
Fixation time, %	11.95	7.40	11.39	11.40	–1.106	0.2689

*The table illustrates the changes in parameters of eye movements in Defectors during the experimental stages: Anonymous and Group stage, Wilcoxon matched pairs test.*

Here, we find increasing dwell and fixation times with no changes in revisits and fixation duration in Cooperators throughout the experiment. We suggest that social domain, which enhances cooperative behavior in Cooperators also made them more attentive to the task (PD) stimuli. Cooperators spend more time in fixations and looking at the PD areas throughout the experimental stages.

Contrary, Defectors’ gaze behavior dynamics had fewer changes during the experiment. Defectors showed no differences in dwell time, fixation count and time, and number of revisits to the areas already scanned. However, the average duration of fixation in Defectors increased through the experimental stages.

## Discussion

Exploration of eye-gaze patterns in human decision processing is a subject of interest for a wide range of scientific disciplines. In this study, we investigated the interrelation of eye movements in the decision process of strategic-based behavior under the influence of a social domain. A large literature in behavioral science emphasizes in the last decades the role of social factors in shaping certain behavioral strategies in various environments (Dal Bo and Frechette, 2018, 2019; Schmitz, 2019; Proto et al., 2020; Peshkovskaya et al., 2021). Moreover, the evidence of social factor influence on a physiological basis of cognitive processes is shown (Do et al., 2019). For better understanding eye-movement patterns’ association with decision strategy, we applied a social psychology minimal group paradigm and the theory of sociality (Lukinova et al., 2014) to enhance participants’ strategic behavior and, particularly, its prosocial aspect.

Generally, prosocial, cooperative behavior is defined as a contributors’ actions that benefit other people, the group, or society (Baumsteiger, 2019; Thielmann et al., 2020). Prosocial behavior can be generated through social interaction between members of the group, accompanied by identification with the group, which shapes an added value of collective interaction (Tajfel et al., 1971; Dasgupta, 2004; Peshkovskaya et al., 2019b). Forcing participants’ strategic behavior with social interaction, we find that the social domain enhances cooperative behavior by a heightening the share of cooperative decisions. To summarize, our study shows that even a brief social interaction significantly improves the level of cooperation, prosocial decisions, and overall cooperative strategy.

Second, we found that eye gaze projected the decision process in strategic-based behavior. In particular, there are certain gaze features for cooperative and non-cooperative strategies. Whereas a number of previous studies reveal that an experiment’s participants approach the visual stimuli selectively and focus their attention on certain areas only (Hristova and Grinberg, 2005; Devetag et al., 2016), we present evidence that selectivity of attention based on strategic-based behavior. We also find that individuals with a highly cooperative strategy pay more attention to a task stimuli and show a more detailed and “careful” approach with a greater number of fixations and frequent gaze returns to the scanned areas. Moreover, our findings confirm the evidence on certain behavioral tendencies in individuals with a highly cooperative strategy to show less interest in non-cooperative decision outcomes. At the same time, participants with a non-cooperative strategy (Defectors) approach visual stimuli in a distinct way. Defectors show long-duration fixations and a low number of gaze returns to the areas already scanned.

Third, several studies suggest gaze time as well as decision time as potentially important parameters for the decision process outcome (Fiedler et al., 2013; Isham and Geng, 2013). Interestingly, we find no differences between total time spent by both Cooperators and Defectors in gazing the payoff matrix at both experimental stages. However, based on our findings, we suggest that the total time an individual spends watching the area of cooperative choice antedated the cooperative decision.

Undoubtedly, understanding human behavior remains a grand challenge across disciplines. In this study, we provide empirical evidence for eye-gaze behavior, decision process, and cooperative strategy interrelation. We emphasize the importance of future research based on state-of-the-art intelligent methods and techniques for better understanding the human decision process associated with strategic experience. We suggest that our findings could help scientists to model sophisticated human decision-making processes to solve real-world problems.

## Data Availability Statement

The datasets generated for this study are available on request to the corresponding author.

## Ethics Statement

The studies involving human participants were reviewed and approved by Tomsk State University Human Subjects Committee. The patients/participants provided their written informed consent to participate in this study.

## Author Contributions

MM contributed to the study design. AP implemented the study, analyzed the results, and wrote the manuscript. AP revised the manuscript. AP and MM equally contributed to the final revision and approved the final version of the manuscript. Both authors contributed to the article and approved the submitted version.

## Conflict of Interest

The authors declare that the research was conducted in the absence of any commercial or financial relationships that could be construed as a potential conflict of interest.

## Supplementary Material

Supplementary Table 1 The differences in eye movement parameters at the Group Game stage.
