# Supplementary material for: Eye Gaze Patterns of Decision Process in Prosocial Behavior
Source: Front Behav Neurosci. 2020 Oct 22;14:525087. doi: 10.3389/fnbeh.2020.525087 (PMC7642209; doi:10.3389/fnbeh.2020.525087)
Supplement: Supplementary Table 1 — The differences in eye movement parameters at the Group Game stage. [file Table_1.docx]

**Table S1.** The differences in eye movement parameters at the Group Game stage.

|  | Cooperators | | Defectors | | Z | p-value |
| --- | --- | --- | --- | --- | --- | --- |
|  | Mean | Median | Mean | Median |  |  |
| Revisits | 0.70 | 0.00 | 0.24 | 0.00 | 2.167 | 0.030 |
| Fixation Count | 2.35 | 2.00 | 1.56 | 1.00 | 2.483 | 0.013 |
| Dwell Time, % | 13.20 | 10.60 | 11.85 | 11.40 | -0.056 | 0.955 |
| Fixation Time, % | 11.88 | 10.45 | 11.39 | 11.40 | -0.406 | 0.685 |
| Average Fixation Duration, ms | 168.72 | 161.25 | 242.95 | 256.00 | -4.247 | 0.00002 |

The table shows the differences between cooperators and defectors in the parameters of eye movements at the Group stage, Mann-Whitney U-Test.
